# MDC-RHT: Multi-Modal Medical Image Fusion via Multi-Dimensional Dynamic Convolution and Residual Hybrid Transformer

**DOI:** 10.3390/s24134056

**Published:** 2024-06-21

**Authors:** Wenqing Wang, Ji He, Han Liu, Wei Yuan

**Affiliations:** 1 School of Automation and Information Engineering, Xi’an University of Technology, Xi’an 710048, China; 2220321215@stu.xaut.edu.cn (J.H.); liuhan@xaut.edu.cn (H.L.); yuanwei@stu.xaut.edu.cn (W.Y.); 2 Shaanxi Key Laboratory of Complex System Control and Intelligent Information Processing, Xi’an University of Technology, Xi’an 710048, China

**Keywords:** medical image fusion, residual hybrid transformer, multi-dimensional dynamic convolution, deep learning

## Abstract

The fusion of multi-modal medical images has great significance for comprehensive diagnosis and treatment. However, the large differences between the various modalities of medical images make multi-modal medical image fusion a great challenge. This paper proposes a novel multi-scale fusion network based on multi-dimensional dynamic convolution and residual hybrid transformer, which has better capability for feature extraction and context modeling and improves the fusion performance. Specifically, the proposed network exploits multi-dimensional dynamic convolution that introduces four attention mechanisms corresponding to four different dimensions of the convolutional kernel to extract more detailed information. Meanwhile, a residual hybrid transformer is designed, which activates more pixels to participate in the fusion process by channel attention, window attention, and overlapping cross attention, thereby strengthening the long-range dependence between different modes and enhancing the connection of global context information. A loss function, including perceptual loss and structural similarity loss, is designed, where the former enhances the visual reality and perceptual details of the fused image, and the latter enables the model to learn structural textures. The whole network adopts a multi-scale architecture and uses an unsupervised end-to-end method to realize multi-modal image fusion. Finally, our method is tested qualitatively and quantitatively on mainstream datasets. The fusion results indicate that our method achieves high scores in most quantitative indicators and satisfactory performance in visual qualitative analysis.

## 1. Introduction

Since different modal medical images have their advantages and limitations, single-modal medical images usually cannot provide comprehensive diagnostic information for clinics. Therefore, the fusion of different modalities of medical images by exploiting their respective advantages has become an important direction of medical image research. In this field, the fusion of structural medical images, e.g., magnetic resonance imaging (MRI), and functional medical images, e.g., positron emission tomography (PET) or single photon emission computed tomography (SPECT), is a research hotspot. MRI, PET, and SPECT are three common medical imaging modes. Specifically, the SPECT/PET provides functional and metabolic information through radionuclide uptake [1], while the MRI provides high-resolution anatomical and soft tissue contrast information [2]. The fusion of SPECT/PET and MRI images can provide images with both morphological and functional information and enable more accurate and reliable location, qualitative analysis, and quantitative analysis of lesions [3]. Therefore, medical image fusion technology is widely used in clinical medicine, which plays an important role in improving the accuracy of diagnosis, optimizing treatment plan, and enhancing the effect of operation.

According to the principles of the traditional fusion methods, they can be divided into four categories: sparse representation-based methods [4], spatial domain-based methods [5], frequency domain-based methods [6], and fuzzy set domain-based methods [7]. Most of the above methods use different image transformation or decomposition techniques to extract multi-scale image features and obtain the expression of images in different spatial dimensions. However, due to the manually designed feature extraction or transformation rules, these fusion methods have limited adaptivity and robustness. Additionally, these methods cannot be trained in an end-to-end manner and are not suitable for complex scenes. Moreover, some effective information may be lost in the process of image decomposition and feature extraction, which makes the fusion method fail to balance between global consistency and local accuracy.

With the rapid development of artificial intelligence, deep learning technology has been widely used in the field of medical image fusion [8]. The deep learning-based methods do not need a manually designed feature extractor and can extract high-level features from the original image data through the multi-layer structure of the deep neural network to capture the potential information of the image [9,10,11]. However, due to the inherent limitations in its mechanism, the deep learning-based fusion methods still have certain defects. (1) Most of the deep learning-based fusion methods using vanilla convolution have limited feature extraction capability. Compared to vanilla convolution, dynamic convolution has a relatively stronger feature characterization capability by aggregating multiple convolutional kernels. The existing dynamic convolution methods focus only on the dimension of the number of convolutional kernels in the kernel space while ignoring the other dimensions, so they have a limited ability to capture contextual cues. (2) The local connectivity and shared weights in the convolutional layer of the deep learning method make it difficult to model long-range dependencies, resulting in a lack of global consistency in the generated images. (3) The medical images usually contain different forms of noise, including artifacts, uneven brightness, and pseudo structures. However, deep learning-based fusion methods often use pixel-level loss functions that do not consider the correlation between pixels, causing the fusion results to be easily interfered with by noise and reducing the quality of the generated images [12].

To overcome the previously mentioned defects and fully unleash the potential of deep learning methods for medical image fusion, this paper proposes a multi-modal medical image fusion method that uses multi-dimensional dynamic convolution (MDC) and residual hybrid transformer (RHT), which is named as MDC-RHT. In the proposed method, the convolutional computation is performed using MDC instead of the conventional vanilla convolution. To effectively capture both the global features and the local details, a RHT module is designed, which integrates channel attention and window-based self-attention mechanisms. The multi-scale approach used in the overall network design highlights the cross-scale properties. Additionally, a loss function is formulated by combining content perception loss and structural similarity loss to improve the resemblance of the fused image to the real image in terms of perception and guarantee that the fused image preserves the structural similarity of the real image. The contributions of this paper are summarized as follows:

1. We propose a novel multi-scale unsupervised fusion network for multi-modal medical images. In order to achieve better fusion, we construct a feature extraction module to acquire more rich information.
2. We propose the use of MDC to comprehensively enhance the image feature extraction from the four dimensions of the convolutional kernels and learn the complementary attention of convolutional kernels in four dimensions to fully capture rich contextual cues.
3. To solve the problem that some transformer mechanisms cannot perfectly achieve cross-window information interaction and result in artifacts in the fused results, the RHT module is designed in this paper. This module can effectively extract global features and enhance the direct interaction of neighboring window features.

The rest of this paper is organized as follows. Section 2 introduces the related work and the motivation, including a brief overview of current medical image fusion methods, a brief introduction to the vision transformer, and an explanation of the motivation of this study. Section 3 provides a comprehensive explanation of the proposed method. The experimental results and analysis are given in Section 4. Section 5 discusses the work of this article. Finally, Section 6 concludes this paper.

## 2. Related Work and Motivations

### 2.1. Multi-Modal Medical Image Fusion Methods

According to the technological principles of image fusion methods, they can be divided into two categories: traditional methods and deep learning-based methods. The traditional methods include sparse representation methods, spatial domain-based methods, frequency domain-based methods, and fuzzy set-based methods. Specifically, the sparse representation-based methods exploit the sparsity characteristic of the source images to realize image fusion by sparse decomposition and coefficient combination [13,14]. The spatial domain-based methods perform pixel-level operations and fusion directly within the spatial domain of the image. Representative spatial domain-based methods include the pyramid transform [15] and the Gaussian pyramid transform [16]. The frequency domain-based fusion methods transform the source images into the frequency domain and utilize the frequency domain characteristics for fusion. The representative methods include wavelet transform [17], curvelet transform [18], non-subsampled contourlet transform (NSCT) [19], and non-subsampled shearlet transform (NSST) [20]. The fuzzy set domain-based methods mainly rely on the concept and operation of fuzzy sets to realize image fusion through blurring, rule building, and defuzzification [21].

The deep learning-based methods do not require manually designed algorithms and rules by using data-driven technology to automatically learn complicated feature representations [22]. Zhang et al. proposed a generalized image fusion framework IFCNN based on convolutional neural networks (CNNs) [23]. The method exploits an attention mechanism to guide the fusion process and improve fusion performance. However, this method only applies linear element-level fusion rules to combine convolutional features. Cheng et al. proposed a memory cell-based image fusion network called MUFusion, which collaboratively supervises the fused image by introducing a novel memory cell architecture that leverages the intermediate output [24]. Liu et al. proposed a novel MIF framework, which integrates the powerful feature representation ability of a deep learning model and the accurate frequency decomposition characteristics of discrete wavelet transform (DWT) [25]. Xu et al. proposed an unsupervised enhancement medical image fusion method EMFusion [26], which evaluates the information content of images by calculating their entropy.

Compared with traditional methods, deep learning-based image fusion methods have made great progress but still have shortcomings. For instance, the convolutional modules used by the current training networks overly focus on extracting local features, and they cannot model long-range dependencies of global information. Meanwhile, convolutional networks are constrained by their local receptive fields, which limits their ability to capture a comprehensive panoramic context. Additionally, due to the lack of attention mechanisms, most of the existing methods cannot effectively extract the correlation between regions and channels. With the excellent performance of transformers on computer vision tasks, an increasing number of transformer-based image fusion methods have been proposed.

### 2.2. Application of Transformer in Computer Vision

The application of transformers in computer vision includes image classification, object detection, semantic segmentation, image generation, etc. The vision transformer method proposed by Dosovitskiy et al. applies transformer to image classification [27]. It abandons the convolution operation in CNN and fully utilizes the attention mechanism in the transformer. Zheng et al. introduced a novel image segmentation method based on the transformer model [28]. By utilizing the self-attention mechanism of the transformer model, the method effectively overcomes the difficulty of establishing contextual connections across large spans. The swin transformer, as proposed by Liu et al. [29], is a hierarchical visual transformer that is designed to effectively capture both local and global information in images by using a moving window.

In terms of multi-modal image fusion, Wang et al. developed a residual swin transformer fusion network called SwinFuse [30] for image fusion. The design of this network fully exploits the capability of the swin transformer to capture global and local feature relationships in image fusion, thus generating more accurate and clear fusion images. Li et al. proposed the DFENet [31] fusion method, which combines transformer and convolutional feature learning to form a dual-branch feature enhancement network. However, the method may face issues of high computational complexity and high memory consumption when processing large-scale image data. Tang et al. presented a multi-modal medical image fusion method called MATR by using a multi-scale adaptive transformer [32]. This method introduces adaptive convolution and an adaptive transformer to extract global complementary contextual information to achieve accurate image fusion.

### 2.3. Motivations

Current image fusion methods based on CNN and transformer have achieved good results, but vanilla convolution or ordinary dynamic convolution still has difficulties in adequately capturing feature context cues. In addition, due to the limitations of its computation principles, ordinary transformers have high computational complexity. Methods such as the swin transformer, which is developed by optimizing an ordinary transformer, can guarantee the quality of the operation while improving the computational speed. However, the shifted window mechanism cannot perfectly realize the cross-window information interaction, and the block effect will appear in the middle of the extracted features. Reference [33] pointed out that the window partitioning mechanism of the swin transformer causes obvious blocking artifacts in the extracted features, indicating that the shifted-window mechanism is inefficient for establishing cross-window connections. References [34,35] suggested that the enhanced connections between windows can improve window-based self-attention methods and reduce artifacts caused by inefficient cross-window connections. Therefore, to solve the above problems, this paper introduces multi-dimensional dynamic convolution into the proposed method. Meanwhile, a novel multi-dimensional attention mechanism and a parallel strategy are employed to learn complementary attentions for convolutional kernels along all four dimensions of the kernel space at any convolutional layer. The MDC fully exploits the potential of dynamic convolutional properties to enhance the efficiency and accuracy of feature extraction. Additionally, the RHT module is designed to combine channel attention and window-based self-attention schemes. It integrates the complementary advantages of global data statistics and powerful local fitting. To overcome the defect of cross-window information interaction, the RHT module also introduces a new overlapping attention module to enhance the interaction between neighboring window features, thereby improving the representation of window self-attention.

Based on the above discussion, this paper proposes the MDC-RHT network, which exploits the advantages of current image fusion methods and solves the problems in these methods. The MDC-RHT network adopts an end-to-end multi-scale network architecture. Its training is performed in an unsupervised manner without manually designing and adjusting the intermediate steps or feature representations, thereby reducing manual intervention and complex parameter tuning. The multi-scale network architecture preserves image details across different scales, which helps to prevent information loss and produce a richer fused image. The loss function employs both content perception (PERCE) loss and structural similarity (SSIM) loss. The PERCE loss can capture several aspects of perceptual content such as image texture, color, and structure, allowing the model to focus on high-level semantic information and global features; the SSIM loss considers three aspects of similarity, namely, brightness, contrast, and structure, and can measure the degree of structural preservation between the fused image and the real image. The combined use of two losses will constrain model training in all directions, producing high-quality fusion results. Additionally, the construction of the MDC and RHT modules is crucial, where the former effectively captures rich contextual cues, and the latter aggregates cross-window information in addition to channel attention and window-based self-attention. This overcomes the limitations of the shift-window mechanism in certain transformers. Finally, the qualitative and quantitative evaluation results demonstrate that our method achieves excellent results in the fusion of SPECT/PET images and MRI images.

## 3. Proposed Method

In this section, the proposed method is introduced in detail. Section 3.1 gives a preliminary description of the overall network architecture of the proposed method. Section 3.2 describes each submodule of the network in detail. The proposed loss function is introduced in Section 3.3.

### 3.1. *Overall Network Framework*

**Figure 1:**
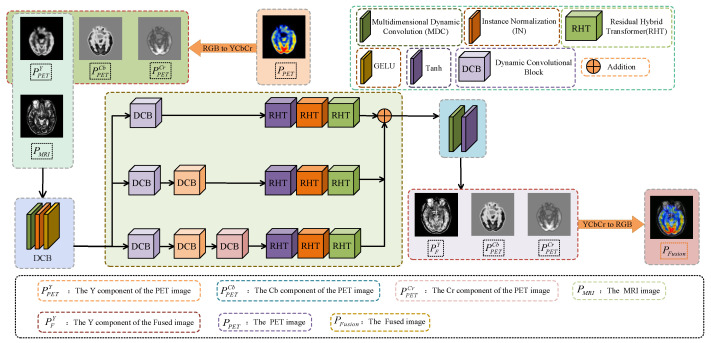
The main framework of MDC-RHT. The SPECT/PET image is decomposed into three components: $P_{PET}^{Y}$, $P_{PET}^{Cb}$, and $P_{PET}^{Cr}$. $P_{PET}^{Y}$ and the MRI image $P_{MRI}$ are sent into the fusion network together to obtain $P_{F}^{Y}$; then, the components $P_{PET}^{Cb}$, $P_{PET}^{Cr}$, and $P_{F}^{Y}$ are used to obtain the final fusion image $P_{Fusion}$ through inversion transform.

As illustrated in Figure 1, the SPECT/PET images contain prominent functional information of tissues and organs. During the fusion process, the functional information, i.e., the color information, should be preserved in the fusion result. However, the MRI image is only a single-channel grayscale image. To implement image fusion, the SPECT/PET image should be transferred from the RGB space to the YCbCr space by using Equation (1). The components Cb and Cr are separated and retained in the chromaticity channel. The component Y represents the luminance channel. Then, the component *Y* of the SPECT/PET image and the MRI image are fed to the end-to-end fusion framework together:

(1) $$\left(\begin{array}{c} Y\\ \mathrm{Cb}\\ \mathrm{Cr} \end{array}\right)=\left(\begin{array}{ccc} 0.299 & 0.587 & 0.114\\ -0.169 & -0.331 & 0.500\\ 0.500 & -0.419 & -0.081 \end{array}\right)\left(\begin{array}{c} R\\ G\\ B \end{array}\right)+\left(\begin{array}{c} 0\\ 128\\ 128 \end{array}\right)$$

(2) $$\left(\begin{array}{c} R\\ G\\ B \end{array}\right)=\left(\begin{array}{ccc} 1.000 & 1.000 & 1.400\\ 1.000 & -0.343 & -0.711\\ 1.000 & 1.765 & 0.000 \end{array}\right)\left(\begin{array}{c} Y\\ Cb-128\\ Cr-128 \end{array}\right)$$

The overall fusion framework is composed of multi-scale modes. Different scale features are extracted through three branches to better preserve contextual interaction information and high-resolution and low-resolution information. The top branch is composed of a dynamic convolutional block (DCB) module and three RHT modules to extract surface features. In the middle branch and the bottom branch, the DCB modules are added layer by layer to extract deep features step by step. Finally, the features of different scales are added and fed into the convolution activation module to obtain the preliminary fusion results. After the fusion process is completed, the chrominance channels Cb and Cr are synthesized into the fused image. The final fusion results can be restored by Equation (2). Through chromaticity separation and restoration, the color information of the original image can be preserved, and the color fidelity is high.

### 3.2. *Description of DCB and RHT*

The DCB module is an important module in the proposed method, which is composed of MDC, instance normalization (IN), and Gaussian error linear units (GELUs). In the image fusion process, the fusion performance depends on whether high-quality image features can be extracted. The fusion of SPECT/PET and MRI images is to integrate the functional information of SPECT/PET images and the structural information of MRI images into a fused image. Therefore, the initial convolutional module needs to have a higher capability for feature extraction to extract more detailed information. Inspired by [36], the MDC is introduced to replace common vanilla convolution and common dynamic convolution.

**Figure 2:**
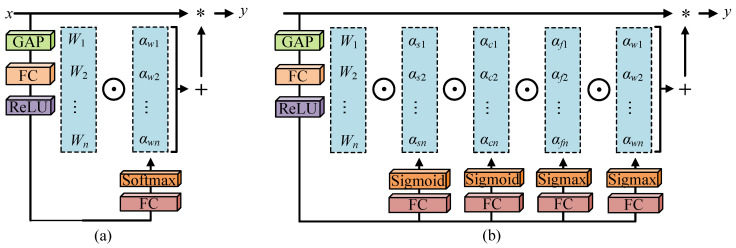
The schematic diagram of dynamic convolution. The standard dynamic convolution is shown in (**a**), while the multi-dimensional dynamic convolution is shown in (**b**). The GAP represents global average pooling, and the FC represents fully connected. * represents convolution operations.

In traditional CNN, each convolutional layer usually learns only one static convolutional kernel. Dynamic convolution can enhance the accuracy of lightweight CNN by learning a set of convolutional kernels and their corresponding attention weights while maintaining an efficient inference process. Nevertheless, the existing dynamic convolution methods, as shown in Figure 2a, only have dynamic characteristics in the dimension of the number of convolutional kernels while ignoring the dimensions of space size, the number of input channels, and the number of output channels. The multi-dimensional attention is introduced in this paper as shown in Figure 2b. Four types of attention mechanisms, i.e., $\alpha_{si}$, $\alpha_{ci}$, $\alpha_{fi}$, and $\alpha_{wi}$, are introduced, corresponding to the spatial dimension of the convolutional kernel, the dimension of the input channel, the dimension of the output channel, and the number of convolutional kernels, respectively. So, the convolutional operation has different responses to different samples. Specifically, $\alpha_{si}$ gives different attention values to the spatial position of each convolutional kernel, $\alpha_{ci}$ gives different attention to the input channel of each convolutional kernel, $\alpha_{fi}$ gives different attention to the output channel of each convolutional kernel, and $\alpha_{wi}$ is used to adjust the weights between different convolutional kernels. These four types of attention mechanisms support parallel computing and gradually act on the convolutional kernel so that the convolutional kernel can make a more dynamic and personalized response to the input samples. The operation of DCB is shown in Equation (3):

(3) $$H_{DCB}^{out}=GELU\left(IN\left(MDC\left(H_{DCB}^{in}\right)\right)\right)$$

where *MDC* represents multiple dynamic convolutions, *IN* represents instance normalization, and *GELU* is an activation function.

**Figure 3:**
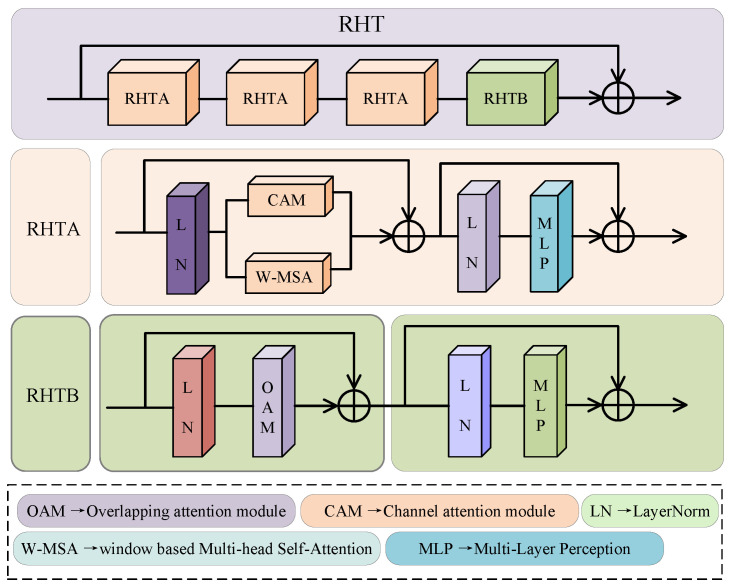
The schematic diagram of the RHT module. The RHT module consists of a residual network with RHTA and RHTB.

To collect multi-resolution feature maps, the overall network architecture uses the multi-scale mode [32]. The research in the literature [33] suggested that a module with channel attention, window attention, and overlapping cross attention can be designed to activate more pixels to participate in the fusion process and strengthen the connection of global context information. Specifically, this paper uses three branches to collect the resolution information of different depths. The top branch consists of one DCB module and three RHT modules, the middle branch consists of two DCB modules and three RHT modules, and the underlying branch consists of three DCB modules and three RHT modules. As demonstrated in Figure 3, the RHT module consists of the RHTA and RHTB sub-blocks connected by residuals. The schematic diagram of RHTA and RHTB is presented below. The RHTA also consists of two addition operations. The first part is a residual module that is composed of Layernorm (LN), window-based multi-head self-attention (W-MSA), and channel attention module (CAM). The second part is a residual module connected by the LN and a multi-layer perceptron (MLP). The operation of RHTA is expressed in Equations (4) and (5):

(4) $$\begin{array}{c} R_{A1}^{out}=CAM\left(LN\left(H_{DCB}^{out}\right)\right)+W-MSA\left(LN\left(H_{DCB}^{out}\right)\right)+H_{DCB}^{out} \end{array}$$

where *CAM* represents the channel attention module, and *W-MSA* represents window-based multi-head self-attention:

(5) $$R_{A}^{out}=MLP\left(LN\left(R_{A1}^{out}\right)\right)+R_{A1}^{out}$$

where *MLP* represents the multi-layer perceptron, and $R_{A1}^{out}$ represents the output of Equation (4).

The RHTB module consists of two addition operations. The first part is a residual module connected by LN and an overlapping attention module (OAM). The second part is a residual module connected by LN and an MLP. The operation of RHTB can be expressed by Equations (6) and (7):

(6) $$R_{B1}^{out}=OAM\left(LN\left(R_{A}^{out}\right)\right)+R_{A}^{out}$$

where *OAM* represents the overlapping attention module.

(7) $$R_{B}^{out}=MLP\left(LN\left(R_{B1}^{out}\right)\right)+R_{B1}^{out}$$

where *MLP* represents the multi-layer perceptron, and $R_{B1}^{out}$ represents the output of Equation (6).

Therefore, the entire RHT module is represented in Equation (8). Finally, the preliminary fusion features are obtained by summing the results of these three branches. After inputting the features into MDC and finally activating the GULE, the final gray fusion image is obtained:

(8) $$R_{RHT}^{out}=R_{B}^{out}\left(R_{A}^{out}\left(R_{A}^{out}\left(R_{A}^{out}\left(H_{DCB}^{out}\right)\right)\right)\right)$$

where $R_{A}^{out}$ and $R_{B}^{out}$ represent the operations of Equations (5) and (7), respectively.

### 3.3. *Loss Function*

The pixel-level difference loss function focuses only on the numerical differences between each pixel, without considering the variations in the human perception of images. Therefore, even if there are minor discrepancies in the noise between the generated image and the real image, the pixel-level loss function may overemphasize these discrepancies. This will reduce the quality of the fused image and limit the robustness and anti-jamming ability of the model to noise. Compared with the traditional pixel-level difference loss function, e.g., mean square error, the perceptual loss and the SSIM loss can better preserve the structural and perceptual features of the image, thereby generating a more real and natural fusion image. The PERCE and SSIM loss functions are more consistent with the characteristics of human perception because they can simulate the high sensitivity of human eyes to the structure and content of the image, leading to fusion images with higher quality. Since the PERCE loss can capture details such as image content perception, and the SSIM loss measures the degree of structure preservation between the fused image and the real image, the total loss function in this paper is designed as follows:

(9) $$L_{all}=L_{PERCE}+L_{SSIM}$$

where $L_{PERCE}$ represents the perceptual loss, and $L_{SSIM}$ represents the SSIM loss. The perceptual similarity measure involves the following steps. Firstly, the feature vector is extracted by a neural network model, and then the Euclidean distance is calculated by mapping in the feature space. The perceptual similarity loss is defined as follows:

(10) $$\begin{array}{cc} L_{PERCE}= & ∥\phi \left(P_{F}^{Y}\right)-\phi \left(P_{MRI}\right){∥}_{2}^{2}\\ \; & +∥\phi \left(P_{F}^{Y}\right)-\phi \left(P_{PET}^{Y}\right){∥}_{2}^{2} \end{array}$$

where $P_{F}^{Y}$ represents the fused gray-scale image, $P_{MRI}$ represents the MRI image, and $P_{PET}^{Y}$ represents the Y component of the SPECT/PET image. For the feature map ϕ, the popular VGG-16 network is used for pre-training. The perceptual will drive the network to generate an image with similar characteristics to the reference image. The algorithm combines superimposed convolution and hierarchical pool to gradually reduce the spatial dimension and extract higher-level features at a higher level.

The PERCE loss can make the model pay attention to high-level semantic information and content perception, but there are still some defects in the structural attribute constraints, so the SSIM loss function is introduced to deepen the learning of structural information such as scene details and structural texture. According to the design principle of the SSIM loss function proposed by [37], the improved loss function is represented as follows:

(11) $$\begin{array}{c} L_{SSIM}=1-SSIM\left(P_{F}^{Y},P_{MRI}\right)+\left(1-SSIM\left(P_{F}^{Y},P_{PET}^{Y}\right)\right) \end{array}$$

where $P_{F}^{Y}$ represents the fused gray-scale image, $P_{MRI}$ represents the input MRI image, and $P_{PET}^{Y}$ represents the Y component of the input color image. SSIM(.) is expressed as follows:

(12) $$SSIM\left(A,B\right)=\frac{\left(2\mu_{A}\mu_{B}+C_{1}\right)\left(2\sigma_{AB}+C_{2}\right)}{\left(\mu_{A}^{2}+\mu_{B}^{2}+C_{1}\right)\left(\sigma_{A}^{2}+\sigma_{B}^{2}+C_{2}\right)}$$

where $\mu_{A}$ and $\mu_{B}$ represent the mean values of *A* and *B*, respectively. $C_{1}$ and $C_{2}$ are constants. $\sigma_{AB}$ represents the covariance of *A* and *B*, and $\sigma_{A}^{2}$ and $\sigma_{B}^{2}$ represent the variance of *A* and *B*, respectively.

## 4. Experiments and Analyses

In this section, the proposed method is compared with some state-of-the-art medical image fusion methods, both quantitatively and qualitatively. Section 4.1 introduces the medical image datasets and the experimental settings in detail. The comparison methods and the quantitative evaluation methods are described in Section 4.2. Section 4.3 demonstrates the experimental results of the proposed method and the comparison methods and analyzes the experimental results in detail.

### 4.1. *Datasets and Experimental Settings*

The Harvard Medical Image Database (https://www.med.harvard.edu/aanlib/home.html, accessed on 8 February 2024) is a database that contains a large number of registered multi-modal medical images. It is used as the training set and test set in the experiment. Specifically, 310 pairs and 40 pairs of MRI and SPECT/PET images are randomly collected from this common dataset as the training set and test set, respectively. Then, the common data enhancement method of random clipping is adopted, and 310 pairs of medical images are clipped to 19,000 patch pairs. In the training process, the learning rate is fixed at 1 × 10^−4^, the batch size is set to 16, the number of epochs is set to 100, and the optimization algorithm is Adam. To facilitate understanding, the MDC in the DCB block in front of the multi-scale network is referred to as MDconv1, the MDC in the multi-scale network is referred to as MDconv2, and the MDC after the multi-scale network is referred to as MDconv3. The detailed parameters of all convolutional operations in the network architecture are listed in Table 1. The code of the proposed method is available at https://github.com/XUTauto/MDC_RHT (accessed on 11 May 2024).

The experimental platform is a computer equipped with a 64-bit Windows 11 operating system, Intel Core i5-12400F CPU, NVIDIA GeForce RTX 3060 Ti GPU, and 16 GB of RAM.

### 4.2. *Comparison Methods and Evaluation Methods*

To demonstrate the advanced performance of the proposed method, it is compared with seven state-of-the-art medical image fusion methods, including MATR [32], DDCGAN [38], EMFusion [26], U2Fusion [39], IFCNN [23], MSDRA [40], and SwinFuse [30]. These methods cover generative adversarial networks, non-end-to-end fusion networks, end-to-end fusion networks, and transformer networks. The codes of these methods are publicly available or provided by their authors. To ensure the accuracy of the experimental results, all the fixed parameters are set according to the values given by the corresponding papers.

To quantitatively analyze the experimental results, nine mainstream evaluation indicators are applied, including mutual information (MI), non-linear correlation information entropy (NCIE), sum of the correlations of differences (SCD), multi-scale structural similarity (MS-SSIM), entropy (EN), visual information fidelity (VIF), Tsallis entropy ($Q_{TE}$), edge-based similarity measure ($Q^{AB/F}$), and gradient-based metric ($Q_{G}$). A detailed description of these indicators is given below.

(1) MI: It describes the dependence of image content by calculating the amount of information shared between two images. The greater the MI value, the stronger the correlation of the information contained in two source images, and the more MI they share [41].
(2) NCIE: It is an information entropy measure to reflect the performance of image fusion, which considers the influence of the non-linear correlation on the fused results [42].
(3) SCD: It computes the correlation differences between the source image and the fused image in different frequency bands [43]. The higher the SCD value, the better the fused result.
(4) MS-SSIM: It is a measure of multi-scale structural similarity, which examines the structural similarity between the source image and the fused image at different scales and reflects the characteristics of the actual visual system [44]. The ideal value of MS-SSIM is 1.
(5) EN: It is a concept used in information theory to measure the uncertainty of data or the amount of information. In the field of image processing, entropy is often used to measure the complexity or information content [45]. The higher the entropy value, the more information of the source image is preserved in image fusion, and the better the effect.
(6) VIF: It describes the statistical feature distribution of the fused image through the dispersion model, which evaluates the fidelity of the visual information between the test image and the reference image according to the similarity and mutual information in the feature space [46]. The higher the VIF value, the better the performance of the fusion method.
(7) $Q_{TE}$: It is an image evaluation index based on information theory, which evaluates the complexity and quality by measuring the non-uniformity and the amount of information [47]. It adjusts the value of parameter *q* to adapt to the non-Gaussian distribution, thereby measuring the complexity and the amount of information and highlighting its sensitivity and adaptability to non-Gaussian distribution.
(8) $Q^{AB/F}$: It provides a detailed assessment of image fusion quality by quantifying the degree to which edge information and important textural details are maintained in the fused image [48]. The ideal value of $Q^{AB/F}$ is 1.
(9) $Q_{G}$: It focuses on the retention consistency and saturation of image edges and details and evaluates fusion performance by comparing the gradient information between the fused image and the source images [49]. The ideal value of $Q_{G}$ is 1.

### 4.3. *Analysis of Experimental Results*

#### 4.3.1. MRI and SPECT

**Figure 4:**
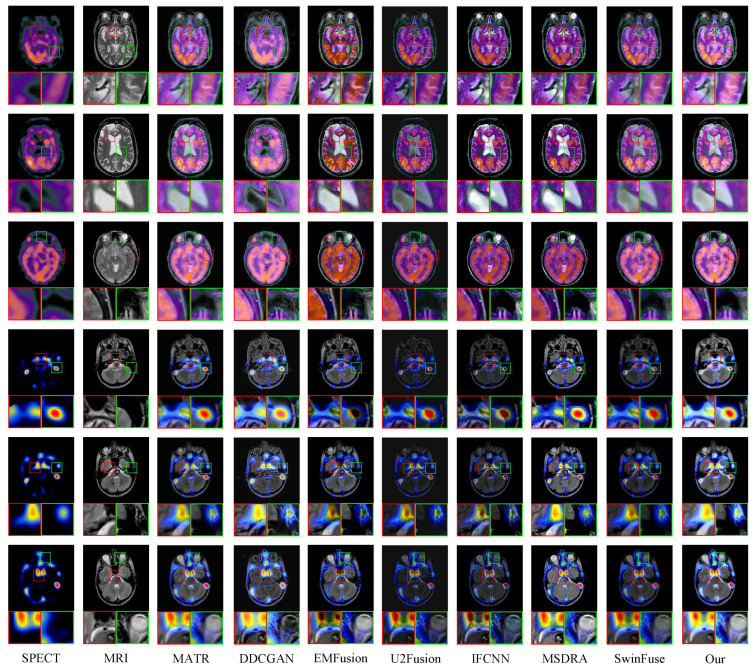
Qualitative evaluation of the proposed MDC-RHT and seven typical and state-of-the-art methods on six representative SPECT and MRI image pairs. From left to right: SPECT, MRI, MATR, DDCGAN, EMFusion, U2Fusion, IFCNN, MSDRA, SwinFuse, and MDC-RHT.

In this section, the proposed method is compared with seven comparison methods on the SPECT and MRI images in terms of visual effect and quantitative analysis. Figure 4 illustrates the fused results of six representative images. To better show the fusion results, two detailed regions are extracted and framed by the red and green boxes, and these magnified regions are put at the left and right bottom of each image. It can be observed from Figure 4 that the DDCGAN and SwinFuse methods can better preserve the functional information from the SPECT images, but they have a poor ability to preserve structural texture information and produce unnecessary artifacts in the fused images. The EMFusion method produces oversharpened fused images due to retaining too much detailed information. The MSDRA method can effectively preserve the color information in the fused images, but the magnified regions show that the fused results of this method contain unnecessary noise and artifacts. The U2Fusion and IFCNN methods have a good ability to preserve detailed information and produce slight distortion in the chromaticity information. The fused images generated by the MATR method show good colors and completely preserve the overall organizational structures, but it can be seen from the local regions that the textural information is still defective. Compared with the above methods, the proposed method performs better in preserving the color information, overall organizational structure, and local texture. It fully retains the functional information of SPECT images and the structural information of MRI images, indicating excellent fusion performance.

**Figure 5:**
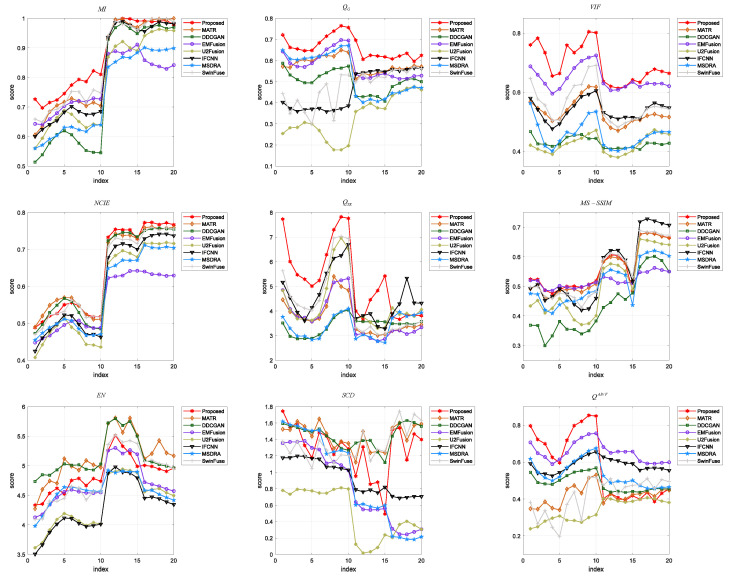
Quantitative comparison of different fusion methods on the SPECT and MRI images in terms of nine objective evaluation indicators.

Then, nine evaluation indicators are employed to quantitatively evaluate the fusion images generated by the proposed method and the comparison methods. Figure 5 shows the trend curves of the nine indicators on 20 randomly selected test images, where the red line represents the proposed method. It can be observed that the proposed method outperforms the other methods in terms of MI, $Q_{G}$, VIF, and $Q_{TE}$. For NCIE, $Q^{AB/F}$, and MS-SSIM, the proposed method has the best values on some test images. For the indicators EN and SCD, the proposed method has relatively poor values compared to some methods. Table 2 lists the average values of each indicator on 20 test images, where the best values are marked in bold and the second-best values are underlined. It can be found that the proposed method has the best average values in terms of MI, VIF, NCIE, $Q_{TE}$, $Q_{G}$, and MS-SSIM, but it has inferior performance in comparison to some methods in terms of EN, SCD, and $Q^{AB/F}$. Generally, the quantitative results demonstrate that the proposed method has better performance in preserving structural information and color information, thereby providing high-quality results for the following visual tasks.

#### 4.3.2. MRI and PET

To verify the generalization ability and robustness of our proposed method, experiments are conducted on MRI and PET images. The PET image refers to the image of metabolism, function, and molecular information in the human body obtained by PET. The MRI image provides high-resolution structural information. The PET functional signals can be located more accurately based on the high-resolution structural image of MRI. Therefore, the fusion of MRI and PET images can provide more accurate focus localization and anatomical location information. In the experiment, seven comparison methods are used, including MATR, DDCGAN, EMFusion, U2Fusion, IFCNN, MSDRA, and SwinFuse.

**Figure 6:**
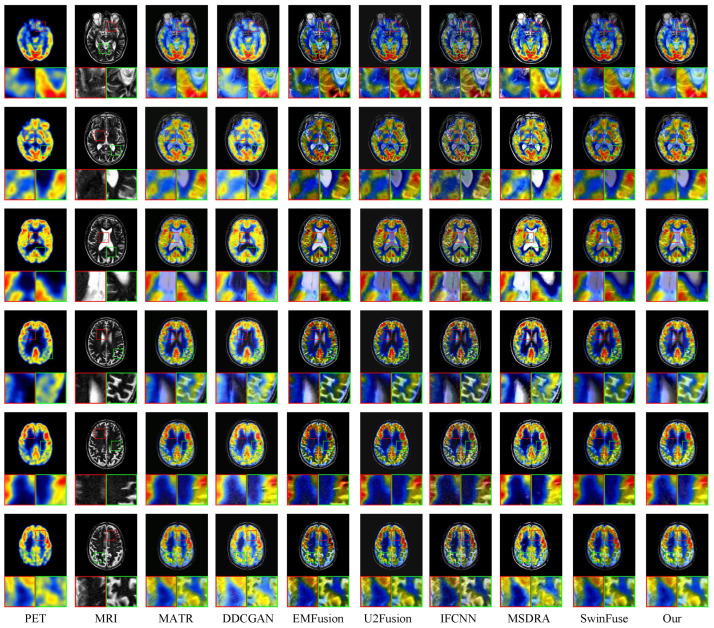
Qualitative evaluation of the proposed MDC-RHT and seven typical and state-of-the-art methods on six representative PET and MRI image pairs. From left to right: PET, MRI, MATR, DDCGAN, EMFusion, U2Fusion, IFCNN, MSDRA, SwinFuse, and MDC-RHT.

Figure 6 shows six pairs of MRI and PET images and the fusion images of eight methods. Two local regions are magnified to better show the fusion performance of different methods. It can be observed that the DDCGAN method can effectively preserve the texture information in the fused image, but the color of the fused images is overexposed. The SwinFuse method generates a fused image with good structural information, but the overall color of the fused image is relatively dark. The MSDRN method preserves the visual features well but produces unnecessary noise. U2Fusion retains chromaticity information well, but the fused images lose some structural information. Compared with the U2Fusion method, the EMFusion method avoids the problem of losing organizational information, but the color of the fused images is relatively dark. From the perspective of the overall visual effect, the fusion images generated by IFCNN and MATR methods have no significant distortion. From the magnified regions, it can be seen that the local regions of the two methods have artifacts. By fully integrating the structural information of MRI images and the functional information of PET images, the proposed method retains the color information of the PET image to the greatest extent and ensures the clarity of detailed textures.

**Figure 7:**
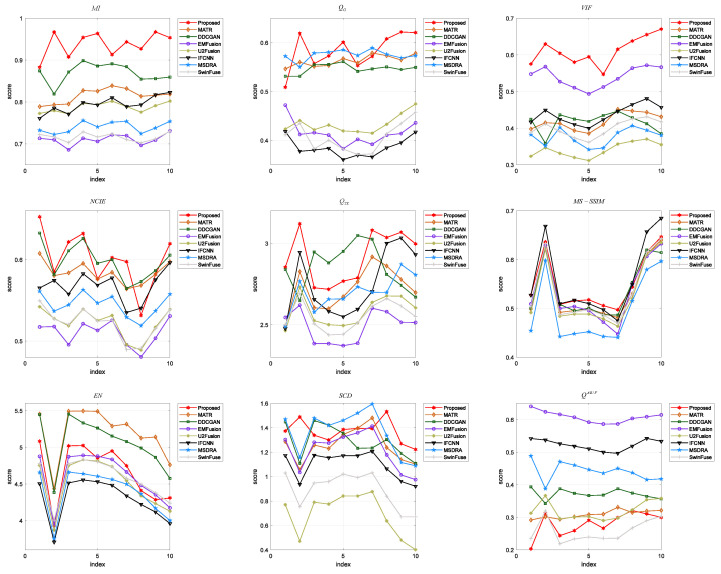
Quantitative comparison of different fusion methods on the PET and MRI images in terms of nine objective evaluation indicators.

The nine evaluation indicators are utilized to make a quantitative analysis of eight fusion methods, and ten pairs of images are selected as the test images. Figure 7 presents a line chart to demonstrate the values and trends of the indicators on each image. Table 3 lists the average value of each indicator for different fusion methods. As shown in Figure 7, our proposed method achieves the best values for all the images in terms of MI and VIF, and it obtains the best average values of MI and VIF. It can be seen from Table 3 that the proposed method achieves the best values of $Q_{G}$, NCIE, $Q_{TE}$, and SCD on some fused images, and it obtains the best average values of these indicators. For the indicators MS-SSIM, EN, and $Q^{AB/F}$, the proposed method has lower values than the other fusion methods. Generally, by effectively integrating the color information of PET images and the structural information of MRI images, the proposed method performs better than the other methods from the perspective of visual quality and quantitative evaluation.

#### 4.3.3. Green Fluorescent Protein and Phase Contrast Image

**Figure 8:**
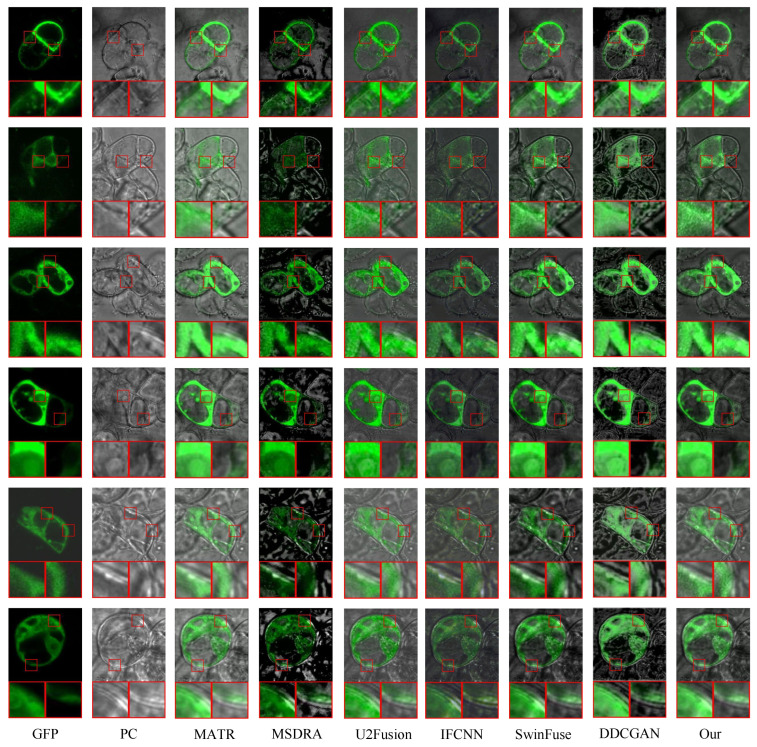
Qualitative evaluation of the proposed MDC-RHT and six typical and state-of-the-art methods on six representative GFP and PC image pairs. From left to right: GFP, PC, MATR, MSDRA, U2Fusion, IFCNN, SwinFuse, DDCGAN, and MDC-RHT.

**Figure 9:**
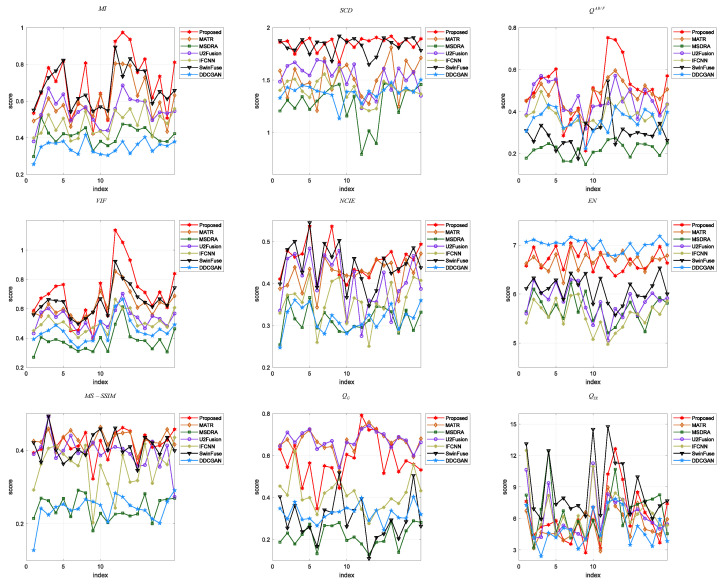
Quantitative comparison of different fusion methods on the GFP and PC images in terms of nine objective evaluation indicators.

Green fluorescent protein (GFP) is a type of protein produced by sea anemones, which has fluorescence characteristics and is widely used in the biomedical field. Phase contrast (PC) imaging is a microscopic technique that uses the optical phase contrast effect to produce light and dark contrast among different tissues or structures of visual cells. The fusion of GFP and PC images can show the cell structure and fluorescence expression signal in the same image so that researchers can observe the cell morphology and gene expression simultaneously [50]. The dataset of GFP and PC images comes from http://data.jic.ac.uk/Gfp (accessed on 3 February 2024). In the experiment, six advanced methods, including MATR, MSDRA, U2Fusion, IFCNN, SwinFuse, and DDCGAN, are taken as the comparison methods. Twenty pairs of GFP and PC images are selected as test images. Figure 8 presents the fused images of different methods. Specifically, the fused image generated by the DDCGAN method has a slightly dark color. Also, it can be observed from the magnified regions that the detailed information is not clear enough. The fused images generated by the SwinFuse, U2Fusion, and MATR methods preserve the color information of GFP images well and the cell structure information of PC images to a certain extent. The MSDRA and IFCNN methods produce fused images with better edge detail information and lower contrast, but the fused images of IFCNN show a blurring effect. Compared with the above methods, our proposed method not only preserves the chromaticity information as much as possible but also fully preserves the details of the tissue structure in the fused images. To objectively evaluate the performance of different fusion methods, nine classical indicators are used. Figure 9 shows the line charts of the nine indicators for different fusion methods on 20 test images. Table 4 lists the average values of the nine indicators for different methods, where the best values are marked in bold, and the second-best values are underlined. It can be seen from Figure 9 that the proposed method has the best values on some test images in terms of MI, SCD, $Q^{AB/F}$, VIF, and NCIE. As listed in Table 4, the proposed method obtains the best average values in terms of MI, VIF, NCIE, SCD, and $Q^{AB/F}$, and it obtains the second-best average values in terms of EN and MS-SSIM indicators. Generally, the proposed method performs well in the fusion of GFP and PC images and achieves better results in terms of both quantitative analysis and qualitative analysis.

### 4.4. Ablation Experiments

#### 4.4.1. *Ablation Experiment of Loss Function*

**Figure 10:**
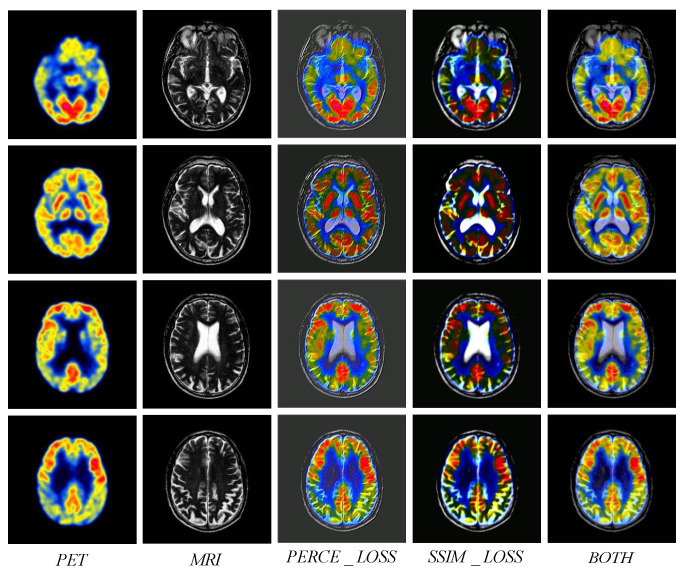
Fusion results under different loss constraints. From left to right: PET and MRI images, only perceptual loss, only SSIM loss, and using both perceptual loss and SSIM loss.

**Figure 11:**
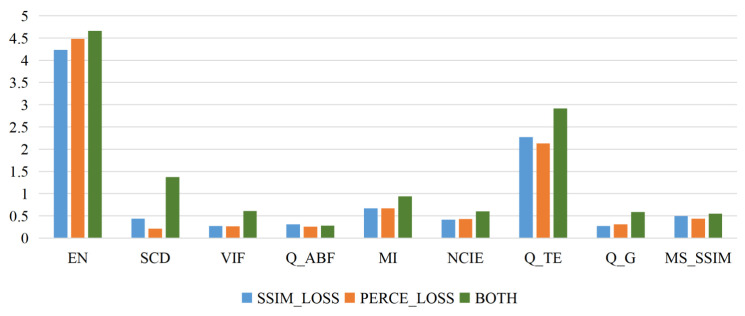
The scores of nine evaluation indicators for fusion results under different loss constraints.

To verify the effectiveness of the loss functions, an ablation experiment is conducted for two loss functions, i.e., $L_{PERCE}$ and $L_{SSIM}$. $L_{PERCE}$ pays attention to the perceptual similarity to enable the model to generate images with more visual realism and perceptual details. Meanwhile, $L_{SSIM}$ pays attention to the structural similarity and enables the model to generate fused images that are closer to the real images. In the ablation experiment, the performance of three methods is compared, i.e., the proposed method with only $L_{PERCE}$, the proposed method with only $L_{SSIM}$, and the proposed method with both $L_{PERCE}$ and $L_{SSIM}$. Figure 10 shows the fused images of the proposed method with different loss functions. It can be found that the fused images generated by the proposed method with only $L_{PERCE}$ have significant differences with the two source images. Although the fused images may preserve some perceptual details, their overall structures contain much noise and many artifacts. The fused images generated by the proposed method with only $L_{SSIM}$ have insufficient visual realism and perceptual details because the model is no longer guided by perceptual features. Compared with the above methods, the proposed method with both $L_{PERCE}$ and $L_{SSIM}$ can generate fused images with better visual features. Figure 11 shows a bar chart of the values of nine indicators for loss functions, where the green bar represents the proposed method with both loss functions. It can be observed that the proposed method with both loss functions has higher values than the proposed method with a single loss function. The experimental results indicate that both $L_{PERCE}$ and $L_{SSIM}$ contribute to higher fusion quality.

#### 4.4.2. *Ablation Experiment of Network Architecture*

**Figure 12:**
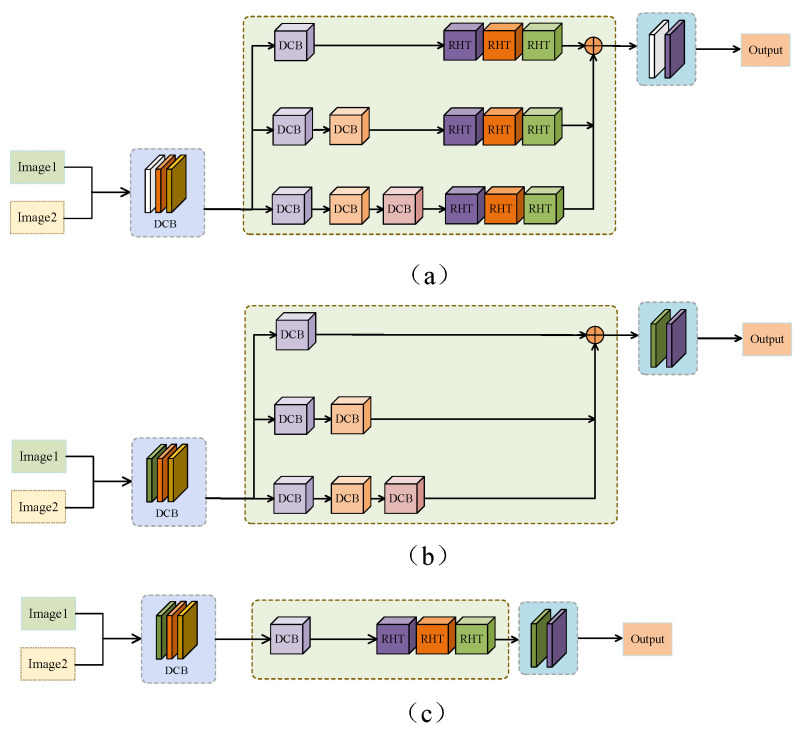
Comparison of the effectiveness of different network architectures. (**a**) Ordinary convolution instead of MDC convolution; (**b**) removal of RHT module; (**c**) single-scale architecture.

In this section, an ablation experiment is conducted to verify the effectiveness of different network architectures, including (1) replacing MDC convolution with ordinary convolution, (2) removing the RHT module, and (3) single-scale architecture as shown in Figure 12a–c. In the first experiment, the MDC convolution is replaced with the ordinary convolution that is the most commonly used in the traditional deep learning algorithm. In the second experiment, the RHT module is removed from the proposed network architecture to verify the effectiveness of our proposed RHT module. The third experiment is conducted to compare the performance of the single-scale network architecture and multi-scale network architecture.

**Figure 13:**
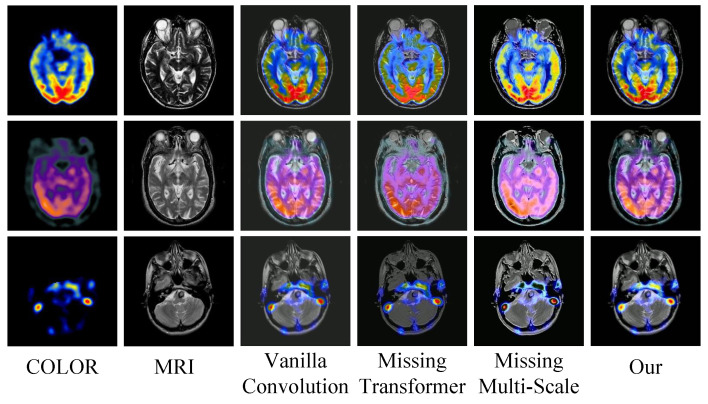
Fusion results under different loss constraints. From left to right: COLOR and MRI images, vanilla convolution instead of ordinary convolution, missing transformer, missing multi-scale, and MDC-RHT.

**Figure 14:**
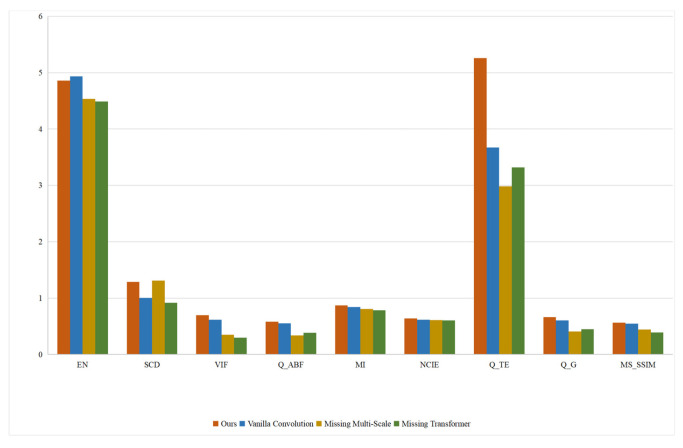
Quantitative evaluation results of the proposed method with different network architectures.

Figure 13 shows the fused images of different network architectures. It can be seen that after using the ordinary convolution instead of the MDC convolution, the fused images tend to be blurred, there is noise and unnecessary artifacts in the fused images, and the local details are not as clear as those of the proposed method. Therefore, it can be concluded that the ordinary convolution is not as capable as the MDC convolution in feature extraction. The proposed method adopts the principle of MDC to fully extract the feature information of the original image from multiple dimensions and channels, which lays a foundation for subsequent operations. It can be observed from the fused images that the proposed method without the RHT module pays too much attention to the local features and fails to establish an effective connection to the global context, so it cannot fully capture the spatial relationship between the images and has poor ability to model the spatial relationship. Hence, the fused images have incoherent local features, lack hierarchy in chromaticity information, and have large areas with sticking colors and distorted texture details. The fused images generated by the single-scale architecture lose much chromaticity information and texture information. The overall resolution of these images is reduced, and there are obvious boundary artifacts in the heterogeneous regions of the fused images. Compared with the single-scale network architecture, the multi-scale network architecture enables the network to handle the differences of different spatial resolutions of the images and learn richer semantic information at different scales. Figure 14 presents the quantitative indicator values of different network ablation experiments. It can be seen that the proposed method obtains better values than the other methods in most evaluation indicators. Through the above experiments and analysis, it can be concluded that the MDC convolution, the RHT module, and the multi-scale network architecture can effectively improve the performance of the proposed method.

## 5. Discussion

The multi-modal medical image fusion method is a key technology for assisting clinical medical diagnosis, which can provide doctors with high-quality medical images. The deep learning-based fusion methods have gradually replaced the traditional fusion methods with their excellent performance and become a research hotspot. In order to obtain fused images with richer information, this paper proposes the MDC-RHT fusion method. This method integrates multi-dimensional dynamic convolution and residual hybrid transformer, which collectively address several critical challenges in multi-modal image fusion.

In Section 4, we verify the performance of the proposed method through three sets of experiments. Better visual quality and higher scores of quantitative indexes indicate the superiority of the fusion algorithm. Figure 4, Figure 6, and Figure 8 shows the fused images of different fusion methods on three datasets, respectively. From a subjective perspective, it can be seen that the fusion results of our method present better visual quality than that of the other methods, especially in terms of color, edge, texture, contour, etc. Figure 5, Figure 7, and Figure 9, and Table 2, Table 3, and Table 4 shows the quantitative results of different methods on three datasets. It can be observed that the proposed method achieves the best results in terms of most indicators compared to the other fusion methods. Moreover, Section 4.4 gives the ablation experiments of loss function and network architecture. The experimental results demonstrate that the loss function and network architecture we have designed are the best.

The remarkable performance of our method observed in the quantitative and qualitative experiments can be attributed to several key innovations in our method. The multi-scale architecture enables the network to fully leverage characteristics across various scales and resolutions, thereby preserving multi-level image details more effectively. As the MDC module with the multi-dimensional attention mechanism allows the convolutional kernels to focus on different dimensions, the DCB module has an excellent ability to capture the local details, thereby efficiently preserving nuanced information from the source images. The RHT module facilitates efficient feature interaction between different windows, which activates more pixels to participate in the fusion process and ensures better preservation of edge and long-range context information. This is crucial for maintaining the structural integrity of the fused images. Additionally, the well-designed loss function ensures that the fused image preserves the complementary information from the source images. Consequently, the excellent performance of the proposed network enhances the quality of image fusion, thereby providing high-quality data support for advanced subsequent medical tasks.

Although the proposed method has achieved good results, there are still some issues that need to be addressed. The high computational complexity caused by the attention mechanisms poses challenges for deploying the proposed method in real-time clinical medical treatment systems. Additionally, considering the stringent requirements for patient privacy and data security in medical applications, enhancing the interpretability and transparency of our model is essential for gaining the acceptance and trust of doctors [51,52].

To address these challenges, the future research will focus on the following aspects: (1) Reducing the computational burden through model pruning and the use of more lightweight networks so that the model is more suitable for clinical applications; (2) employing thermal maps and the other techniques to enhance the interpretability of neural networks, thereby improving the transparency and understandability of the model; and (3) developing a more general fusion network to enhance the generalization capabilities of the model, which adapts to more complex fusion scenarios across different fields [53,54].

## 6. Conclusions

This paper proposes a multi-scale fusion network combining multi-dimensional dynamic convolution and residual hybrid transformer for the fusion of multi-modal medical images. Comprehensive experiments are conducted on three representative datasets. Compared with the state-of-the-art methods, our proposed method achieves higher quantitative scores and better visual quality as indicated by the experimental results and analysis. This study provides important theoretical support for multi-modal medical image fusion based on deep learning.

## Figures and Tables

**Table 1:** The detailed parameters of convolutional operations in the proposed network architecture.

Layers	Size	InC	OutC	Activation
MDConv1	3	2	3	Gelu
MDConv2	3	3	3	Gelu
MDConv3	1	3	1	Tanh

**Table 2:** The average values of the nine evaluation indicators of different fusion methods on 20 pairs of SPECT and MRI images. The best value is marked in bold, and the second best value is underlined.

Methods	MI	$Q_{G}$	VIF	NCIE	$Q_{\mathrm{TE}}$	MS-SSIM	EN	SCD	$Q^{\mathrm{AB}/F}$
Our	**0.8723**	**0.6622**	**0.6954**	**0.6413**	**5.2589**	**0.5626**	4.8604	1.2886	0.5816
MATR	0.8386	0.5798	0.5315	0.6408	3.7351	0.5559	**5.1495**	1.4417	0.4190
DDCGAN	0.7667	0.5005	0.4285	0.6318	3.4257	0.4358	5.1449	**1.4492**	0.4856
EMFusion	0.7801	0.5772	0.6442	0.5578	3.7254	0.5194	4.6963	0.8314	**0.6535**
U2Fusion	0.7822	0.3315	0.4248	0.5806	4.2972	0.5071	4.3542	0.4949	0.3385
IFCNN	0.8177	0.4626	0.5388	0.6006	4.5457	0.5582	4.2905	0.9350	0.5837
MSDRA	0.7447	0.5347	0.4559	0.5838	3.3776	0.5107	4.5847	0.9134	0.5330
SwinFuse	0.8467	0.4771	0.5603	0.6292	4.3138	0.5523	4.8261	1.3442	0.4176

**Table 3:** The average values of nine evaluation indicators of different fusion methods on 10 pairs of PET and MRI images. The best value is marked in bold, and the second best value is underlined.

Methods	MI	$Q_{G}$	VIF	NCIE	$Q_{\mathrm{TE}}$	MS-SSIM	EN	SCD	$Q^{\mathrm{AB}/F}$
Our	**0.9381**	**0.5835**	**0.6111**	**0.6003**	**2.9159**	0.5516	4.6621	**1.3702**	0.2807
MATR	0.8151	0.5633	0.4187	0.5840	2.7211	0.5364	**5.2007**	1.2537	0.3093
DDCGAN	0.8695	0.5467	0.4169	0.5977	2.8561	0.5391	5.0544	1.2863	0.3717
EMFusion	0.7105	0.4149	0.5396	0.5101	2.4905	0.5348	4.5955	1.2161	**0.6081**
U2Fusion	0.7875	0.4332	0.3412	0.5225	2.5824	0.5283	4.4996	0.6886	0.3201
IFCNN	0.7942	0.3854	0.4371	0.5674	2.7474	**0.5597**	4.2928	1.0936	0.5226
MSDRA	0.7402	0.5749	0.3759	0.5449	2.6961	0.4972	4.3914	1.3646	0.4411
SwinFuse	0.7166	0.4061	0.3997	0.5221	2.5534	0.5358	4.5616	0.8917	0.2578

**Table 4:** The average values of nine evaluation indicators of different fusion methods on 20 pairs of GFP and PC images. The best value is marked in bold, and the second best value is underlined.

Methods	MI	SCD	$Q^{\mathrm{AB}/F}$	VIF	NCIE	EN	MS-SSIM	$Q_{G}$	$Q_{\mathrm{TE}}$
Our	**0.7009**	**1.8493**	**0.5025**	**0.7023**	**0.4543**	6.6943	0.4194	0.5751	6.2182
MATR	0.5966	1.5132	0.4825	0.6284	0.4226	6.6818	**0.4284**	0.6653	5.3614
MSDRA	0.6734	1.8268	0.2972	0.6486	0.4432	6.0933	0.4103	0.2981	**8.8552**
U2Fusion	0.4103	1.2728	0.2171	0.3791	0.3133	5.6864	0.2417	0.2181	6.5652
IFCNN	0.5468	1.5271	0.4567	0.5286	0.4021	5.8423	0.3945	**0.6706**	6.3411
SwinFuse	0.4757	1.4068	0.3851	0.5061	0.3561	5.5558	0.3520	0.4138	6.5152
DDCGAN	0.3471	1.3834	0.3575	0.4522	0.3159	**7.0058**	0.2399	0.3241	5.0221

## Data Availability

Publicly available datasets were analyzed in this study.
